# Peripheral overconfidence in a scene categorization task

**DOI:** 10.1167/jov.25.10.2

**Published:** 2025-08-01

**Authors:** Nino Sharvashidze, Matteo Toscani, Matteo Valsecchi

**Affiliations:** 1 Dipartimento di Psicologia, Università di Bologna, Bologna, Italy; 2 Psychology Department, Bournemouth University, Poole, UK

**Keywords:** metaperception, confidence, peripheral vision, foveal vision, scene categorization

## Abstract

Our ability to detect and discriminate stimuli differs across the visual field. Does metaperception (i.e., visual confidence) follow these differences? Evidence is mixed, as studies have reported overconfidence in peripheral detection tasks and underconfidence in a peripheral local orientation discrimination task. Here, we tested whether overconfidence can arise in a task that aligns with the strengths of peripheral vision: rapid scene categorization. In each interval, our participants viewed a scene only in the periphery (scotoma) or only in the center (window) and categorized it (desert, beach, mountain, or forest). Subsequently, they indicated the interval for which they were more confident in their judgment. Task difficulty was manipulated by varying the scotoma and window size. Accuracy decreased with the increasing size of the scotoma and increased with the increasing size of the window. We computed the probability of higher confidence in the periphery as a function of the expected performance difference between the two conditions. Participants’ points of equal confidence were systematically shifted toward higher central perceptual performance, indicating that higher visibility in the center was needed to produce matched perceptual confidence and demonstrating overconfidence in the periphery. This suggests that changing the task from local orientation discrimination to global scene categorization (i.e., a task where peripheral vision outperforms foveal vision) reversed the metaperceptual bias. Periphery is suited for detecting objects and processing global information, but not for discriminating fine details or local features. Metacognitive judgments seem to follow these inherent capabilities and constraints of peripheral vision.

## Introduction

Perception is not uniform across the visual field. The fovea, a central region with the highest density of cone photoreceptors and therefore maximum visual acuity, processes only a small portion of the visual input (Curcio, Sloan, Kalina, & Hendrickson, 1990). The rest of the information is processed in the periphery, where the ability to perceive fine detail declines (Anstis, 1974; Pointer & Hess, 1989), position uncertainty increases (e.g., Michel & Geisler, 2011), contrast and high spatial frequency sensitivity declines (Rovamo, Virsu, & Näsänen, 1978), and processing is further impaired by crowding (Bouma, 1970) (for reviews, see Rosenholtz, 2016; Rosenholtz, 2020; Strasburger, Rentschler, & Jüttner, 2011). Despite these deficits, as Rosenholtz (2016) puts it, “If we actually had to rely on multiple foveal views, our vision would be far worse.” We rely on the periphery to guide our eye movements, to direct the fovea to the areas of interest (e.g., Nuthmann, 2014). Yet, even in their absence, observers demonstrate the ability to rapidly and accurately recognize scenes (e.g., Greene & Oliva, 2009; Loschky et al., 2007). Although scene gist recognition is to some extent an artificial task, as we rarely identify the gist of scenes explicitly in everyday life, it nonetheless mainly relies on global processing, a core function of peripheral vision (e.g., Larson & Loschky, 2009; Trouilloud et al., 2020). Larson and Loschky (2009) tested scenes with either only the central or the peripheral regions visible. They found that, although foveal vision is more efficient for gist recognition, peripheral vision contributes more overall, likely due to its larger viewing field (see also Geuzebroek & van den Berg, 2018; Wang & Cottrell, 2017). While perception varies between the periphery and the fovea, with perceptual efficiency depending on the specific task, are we consciously aware of these differences? Evidence regarding how accurately our metaperception (i.e., our beliefs about our percepts) tracks the perception across the visual field is mixed, so are the methods used to investigate this question (for a review on metaperception, see Mamassian, 2016).

One possibility is that confidence directly tracks perceptual performance; that is, the more accurate the perception across the visual field, the higher the associated confidence. However, although observers typically show some level of metacognition (reviewed below, e.g. Kim & Chong, 2024; Pruitt, Knotts, & Odegaard, 2024), numerous studies have shown biased confidence judgments. For example, overconfidence in peripheral vision has been observed in redundancy-masking paradigms, despite a decrease in accuracy (Yildirim & Sayim, 2022). A line of research, using signal detection theory models of confidence, has reported several further cases of biased metacognition. The data show more liberal detection criteria in the periphery, associated with higher confidence, as such a criterion leads to a higher likelihood to report peripheral stimuli as present when they are absent (e.g., Li, Lau, & Odegaard, 2018; Rahnev et al, 2011; Solovey, Graney, & Lau, 2015). In the study by Odegaard, Chang, Lau, and Cheung (2018), in addition to reporting the presence of the stimulus, observers were explicitly asked to rate their confidence. The results showed overconfidence in discrimination judgments and liberal bias in detection judgments in crowded compared to uncrowded conditions. Notably, all of these results have been taken as support for the “inflation”—the phenomenological richness of vision (at least partially) explained by inflated peripheral perception (Cohen, Dennett, & Kanwisher, 2016; Knotts, Michel, & Odegaard, 2020; Knotts, Odegaard, Lau, & Rosenthal, 2019; for an example of inflation in unattended peripheral stimuli, see also Tian et al., 2024).

Winter and Peters (2022) investigated how the knowledge and use of noise statistics across the visual field influence metaperceptual judgments. They found that models incorporating accurate empirical noise priors for both the fovea and the periphery do not produce metaperceptual biases. In contrast, the observed increase in peripheral confidence could be explained by a model in which the perceptual system relies on an incorrect prior about the shape of the noise distribution. Boundy-Singer, Ziemba, and Goris (2023) proposed that confidence arises from a noisy internal estimate of decision reliability, with the precision of this estimate varying across conditions. Interpreting peripheral overconfidence within their framework, increased meta-uncertainty in peripheral vision can reduce the precision of this estimate, leading to inflated confidence despite reduced accuracy.

Interestingly, a more recent study by Pruitt et al. (2024) examined biases across various horizontal eccentricities and found stronger support for the alignment of confidence judgments with task performance (i.e., unbiased metacognition) rather than overconfidence. Additionally, the detection decision criteria varied, being more liberal at medium eccentricities and more conservative at smaller and larger eccentricities. This aligns with other studies that have found some degree of alignment between perceptual performance and confidence. For instance, observers showed partial awareness of their metacognitive deficits in unattended peripheral areas when processing color stimuli (Hawkins et al., 2022) and for faces along the horizontal meridian (Kim & Chong, 2024). In a degraded face localization task, Kim and Chong (2024) found that, although participants recognized the decline in perceptual resolution with increasing eccentricity, they were less aware of the fact that their performance degraded to a larger extent as a function of eccentricity along the vertical meridian as compared to the horizontal meridian.

Another example of the dissociation between confidence and perceptual performance comes from a study on foveal scotopic scotoma (Gloriani & Schütz, 2019). Under low-light, scotopic conditions, the fovea, which lacks rod receptors, exhibits a central blind spot that typically goes unnoticed due to perceptual filling-in. In the study by Gloriani and Schütz (2019), observers viewed striped gratings that were either continuous or had a central discontinuity falling within the scotoma. As expected, in the first experiment, observers failed to detect central discontinuities when they fell within the scotoma. In the second experiment, observers were asked to choose which stimulus to judge, to reveal their perceptual confidence. They consistently preferred to judge the less eccentric stimulus and reported continuity when both stimuli were continuous and even when the discontinuity fell within the scotoma. Their preference for judging the peripheral stimulus was only increased when the peripheral stimulus contained the discontinuity. The same pattern of results was obtained under photopic conditions. The authors concluded that the scotopic scotoma is not accounted for during perceptual decision-making and that confidence is assessed at a processing stage where information about the underlying photoreceptor type is lost and perceptual filling-in is complete. Relying on central vision under scotopic conditions is suboptimal, as the percept is not veridical but instead filled in; yet, observers still tend to trust it more than veridical peripheral information. It is worth noting that, although the central and peripheral stimuli were clearly suprathreshold (i.e., they were not matched in terms of visibility), the finding that observers consistently chose to judge the central stimulus, under both scotopic and photopic conditions, is suggestive of peripheral underconfidence (for further striking examples of dissociation between perception and confidence, see Michel, Gao, Mazor, Kletenik, & Rahnev, 2024).

Toscani, Mamassian, and Valsecchi (2021) directly compared central and peripheral vision to investigate whether confidence differed when perceptual performance was matched. They adjusted stimulus difficulty individually for each participant for central and peripheral presentations to ensure similar levels of discrimination performance. In their Gabor orientation discrimination task, peripheral vision was associated with underconfidence (see also Knotts, Lee, & Lau, 2019). These findings suggest that, even when perceptual performance is equated across the visual field, confidence bias persists, with peripheral signal being treated as less reliable during a local orientation discrimination judgment.

This raises the question of whether this is a default preference for foveal over peripheral vision or whether the preference is task dependent. Odegaard and Lau (2016) argued that the nature of the decision—specifically, how fine-grained or detailed the task is—should be taken into account when interpreting confidence judgments in peripheral vision. Tasks such as local orientation discrimination or orientation misalignment discrimination in gratings require fine detail vision and precise local processing, functions for which the visual system inherently prioritizes foveal input. Put simply, no one would rely on peripheral vision to thread a needle. In this study, we asked whether tasks in which peripheral vision outperforms central vision can reverse peripheral underconfidence. We selected a task that requires global/coarse instead of local/fine-detail processing: scene categorization. The reasoning is that, if the visual system holds a prior favoring peripheral vision for global scene processing, then confidence judgments should reflect this.

With a confidence forced-choice task (Mamassian, 2020), as in Toscani et al. (2021), we tested scene categorization accuracy and metaperception in a paradigm similar to that of Larson and Loschky (2009). Each trial consisted of two intervals; in each interval, a scene was shown either only in the periphery (scotoma condition) or only in the center (window condition), and for each interval the observers provided a scene categorization response. After the second interval, participants indicated the interval for which they were more confident in their categorization. Task difficulty was varied by varying scotoma size (more peripheral information with a smaller scotoma) and window size (more central information with a larger window). To anticipate our main result, we found that changing the task from orientation discrimination to scene gist categorization also changed observers’ response patterns from underconfidence to overconfidence in the periphery.

## Methods

### Participants

We estimated the required sample size by calculating the effect size from the metacognitive bias results in Experiment 1 of Toscani et al. (2021) (Cohen's *d* = 2.274). A power analysis conducted using G*Power 3.1 (Faul, Erdfelder, Lang, & Buchner, 2007) indicated that a sample size of five participants would be enough to detect the expected effect with a power of 0.95 and a significance level of 0.05. To ensure robust results, we planned to recruit 12 participants. A total of 13 naïve participants volunteered to participate in the experiment without compensation. One participant was excluded due to the inability to keep fixation. The exclusion resulted in a dataset of 12 participants (mean age, 24.43 ± 4.94 years; range, 20–35 years; three males). They reported normal or corrected-to-normal vision and provided written informed consents. All procedures were in accordance with the tenets of the Declaration of Helsinki and were approved by the Bioethics Committee of the University of Bologna (protocol 0122496). The study was not preregistered.

### Equipment

The stimuli were presented on a S2522HG monitor (size, 54.5 × 30.2 cm; resolution, 1920 × 1080 pixels; refresh rate, 60 Hz; Dell Technologies, Round Rock, TX). The viewing distance was 56 cm, which resulted in 34.95 pixels per 1 degree of visual angle (dva) on the vertical axis. Luminance values of the monitor were 0.6, 22.1, and 124.2 cd/m^2^ for black (0), gray (123), and white (255) RGB values. The stimuli were presented using Psychophysics Toolbox 3.0.19 (Brainard, 1997; Pelli, 1997) with Psignifit 4.0 (Schütt, Harmeling, Macke, & Wichmann, 2016) in MATLAB R2023a (MathWorks, Natick, MA). Gaze position was recorded with the Tobii Eye Tracker 4C (Tobii Gaming, Danderyd, Sweden), sampled at 90 Hz, and was used to ensure participants were fixating at the center of the screen. The eye tracker was controlled using custom-made scripts. A nine-point calibration was performed at the beginning of the experiment. Head movement was minimized with a chin rest. Participants responded using a standard keyboard (a, d, h, and k keys for scene categorization and c and b keys for confidence judgment). The assignment of category keys and categories was randomized for each participant, and the confidence response keys were fixed (c for more confidence in the first interval, b for more confidence in the second interval).

### Stimuli

All stimuli were presented on a gray background (RGB value = 128; luminance value = 24.04 cd/m^2^). The fixation target was a combination of a bull's eye and a crosshair (Thaler et al., 2013). The radius of the outer circle of the fixation target was 10.49 pixels (0.3 dva). The cue was a circular ring (1 pixel wide) displayed around the fixation target. The radius of the scotoma cue was 400 pixels (11.44 dva), and the radius of the window cue was 20 pixels (0.57 dva). The fixation target and cue had the same color, which was determined by varying the luminance and red–green components of an approximate DKL color vector on each trial (Derrington, Krauskopf, & Lennie, 1984). This was done to minimize the build-up of afterimages. Notice that we did not measure the chromaticities and luminance of the monitor RGB primaries and resorted to standard RGB to estimate the DKL-to-RGB conversion. The four image categories were beach, desert, forest, and mountain. The images were sourced from the SUN Database (Xiao, Hays, Ehinger, Oliva, & Torralba, 2010) and Flickr. Prior to use, they were equalized for mean luminance of 19.14 cd/m^2^ and contrast *SD* of 1.22 cd/m^2^, as in Loschky et al. (2007).

**Figure 1.**
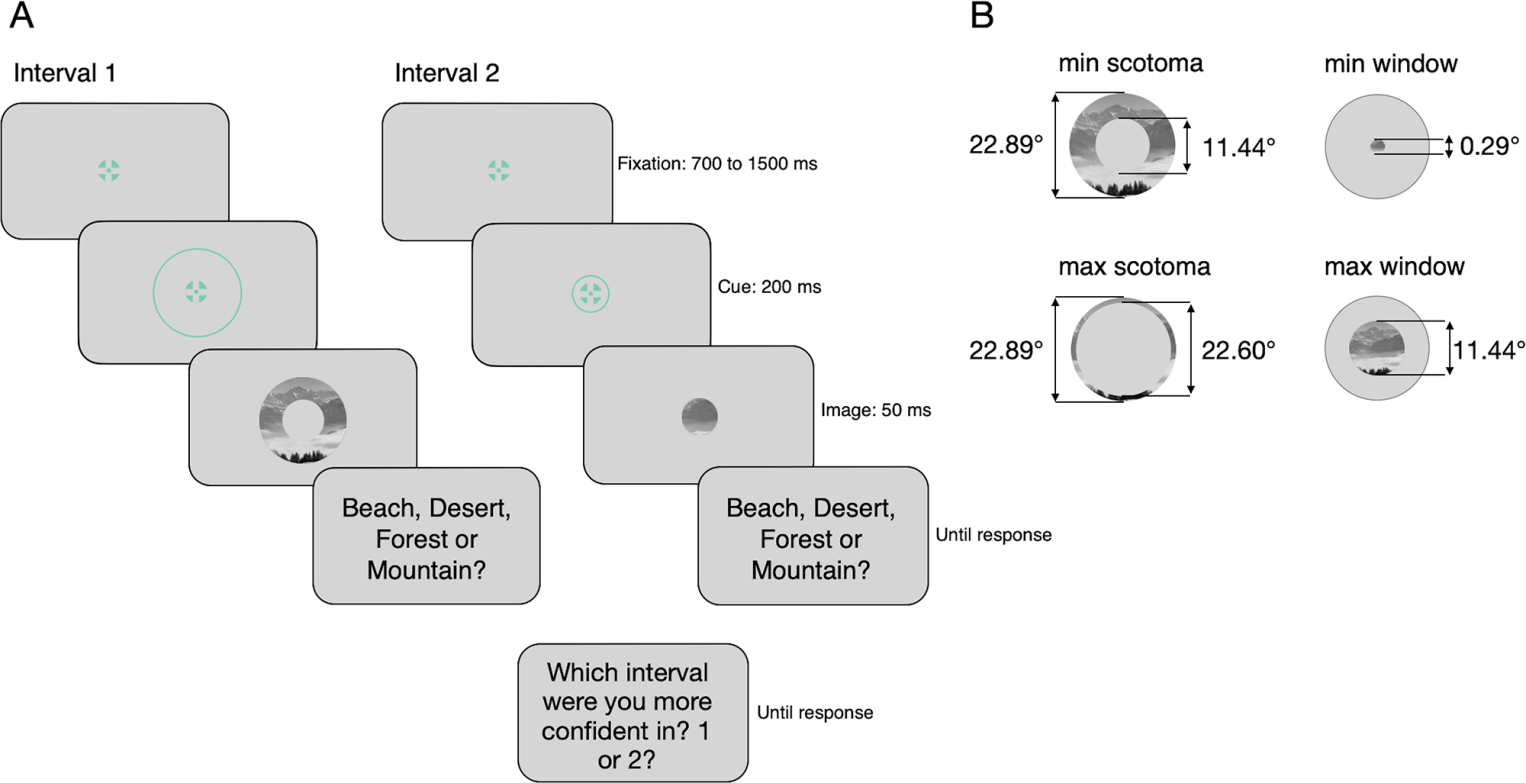
Trial procedure and stimuli. (**A**) Participants first fixated on the fixation target of the first interval for 700 to 1500 ms, followed by a 200-ms cue indicating the condition. The image was then presented for 50 ms, after which participants made a categorization decision. The fixation target reappeared for the second interval, followed by the cue, image presentation, and a second categorization decision. After both intervals, participants made a confidence judgment. Response times were not constrained, and the interval–condition assignment was counterbalanced. Although the same image is shown in both intervals here, different images were used in the experiment. (**B**) An approximate visual representation of the smallest and largest scotoma and window diameters with the corresponding degree of visual angle values. In both conditions, the rest of the screen was gray.

In the *scotoma* condition, only peripheral information was available; in the *window* condition, only central information was available. The outer boundary of the image was fixed at the radius of 400 pixels (11.44 dva) for both conditions, with the area beyond this boundary consistently gray. In the scotoma condition, the central part of the image was not visible (i.e., was gray). The minimum and maximum radii for the window were 5 and 200 pixels, for scotoma – 200 and 395 pixels (Figure 1B). The smallest window of 5-pixel radius resulted in only the central 10-pixel-diameter patch (0.29 dva) of the image being visible and the rest of the screen being gray. The largest window of 200-pixel radius resulted in the 400-pixel-diameter patch (11.44 dva) of the image being visible and the screen beyond that being gray. The smallest scotoma of 200-pixel radius resulted in the whole image (outer boundary at 400 pixels) being visible except for the central area of 400-pixel diameter (11.44 dva), which was gray. The largest scotoma of 395 pixels left only a thin ring (0.29 dva) visible; everything beyond was gray.

### Procedure

Each trial consisted of two scene categorization and one confidence judgments (Figure 1A). The trial began with a fixation target in the center of the screen, which was presented for a variable period of time between 750 and 1500 ms. A circular cue appeared around the fixation target 400 ms before the fixation target offset for 200 ms, indicating the upcoming condition: window (small cue) or scotoma (large cue). The conditions were cued to ensure that attention was distributed equally between the two conditions. Participants were instructed to maintain fixation at the center of the screen throughout the trial. If the gaze position moved more than 2 dva away from the fixation target, they were warned to keep fixation on the target and the trial was repeated later. After the fixation target offset, the image appeared for 50 ms. In each trial, the image was randomly selected from the dataset of 50 images per category. After the first interval, participants categorized the scene by pressing a key to select one of four category options. The response options and corresponding keys were displayed on the screen. This was followed by the reappearance of the fixation target, the cue, the presentation of the second image, and a categorization response. After both categorization responses, participants indicated the interval for which they were more confident in their judgment.

The sizes of the scotoma and window were varied. In the initial phase of the experiment there were five fixed size values for each condition: for the window condition, 5, 53.75, 102.5, 151.25, and 200 pixels; for the scotoma condition, 200, 248.75, 297.5, 346.25, and 395 pixels. All pairwise combinations of initial fixed window and scotoma values were repeated across four categories (5 × 5 × 4). Half of the trials were assigned to scotoma–window order and the other half to window–scotoma order. This resulted in 100 randomized trials for the initial fitting. After the initial fitting, the scotoma and window sizes were defined based on the sampled difficulty levels. Starting after 100 trials and after each trial, a cumulative Gaussian was fitted to the proportion correct responses as a function of the size separately for the window and scotoma conditions, using the *psignifitFast* function (without guess and lapse rates). Performance for both intervals was sampled separately and uniformly from a range between 0.1 and 0.9. The sizes required to achieve the sampled performance levels were determined from the fits with the unscaled *getThreshold* function and were forced to not exceed the size limits of each condition. Ten repetitions of all combinations of interval category and condition order were run after the initial 100 trials, resulting in 320 additional trials (4 × 4 × 2 × 10). In total, the experiment consisted of 420 trials. It was run in one session with five breaks and lasted approximately 1.5 to 2 hours.

### Analysis

#### Categorization accuracy

We expected that the categorization accuracy would improve with increasing peripheral and central information, with more peripheral information corresponding to a smaller scotoma size and more central information corresponding to a larger window size. Cumulative Gaussian functions were fitted to the proportion of correct responses as a function of scotoma size and window size in pixels. Notice that the function has a negative slope in the scotoma condition. Data from all 420 trials were used.

#### Metacognitive bias

To analyze the metacognitive bias, we looked at the relationship between the expected performance difference between the two conditions and the probability of choosing the scotoma interval as higher confidence. To assess performance difference in each trial, we first calculated the proportion of correct responses to be expected for each condition separately based on the window and scotoma sizes and the respective psychometric fits. The proportions were then converted to *z*-scores, similar to Toscani et al. (2021). The difference in the *z*-scores between the scotoma and window conditions was calculated by subtracting the window *z*-score value from the scotoma *z*-score value. The data from the individual trials were binned, and a cumulative Gaussian function (with four parameters, including lapse and guess rates) was fitted to the responses with higher confidence in scotoma as a function of the *z*-score difference values. The points of equal confidence (PECs) could be estimated. A PEC value of 0 indicates no metacognitive bias. Negative PEC values reflect overconfidence in scotoma, where higher window performance is required to match the confidence levels in scotoma. Conversely, positive PEC values indicate overconfidence in the window, where higher scotoma performance is needed to match the confidence levels in the window.

#### Metacognitive sensitivity

To ensure that our participants were able to provide metacognitive judgments that were related to the perceptual difficulty of the trial, rather than simply using window and scotoma sizes as cues to confidence, we decided to compute metacognitive sensitivity. To this aim, we compared participants’ perceptual sensitivity between high- and low-confidence choices. Cumulative Gaussian functions were fitted to the data, split according to which trial interval was selected for higher confidence. High metacognitive sensitivity is reflected in differences between the 50% thresholds for high confidence and low confidence choices. In the case of scotoma, high-confidence trials should have had higher thresholds, as higher threshold values indicate better peripheral perceptual sensitivity. In the case of window, high-confidence trials should have shown lower thresholds, as lower thresholds indicate better central perceptual sensitivity. No threshold differences suggest a lack of metacognitive sensitivity, indicating that participants’ confidence decisions were not based on their accuracy.

### General data analysis

For the data analysis, all psychometric functions were fitted with the Psignifit 4.0 (Schütt et al., 2016) in MATLAB R2022a. Further statistical analyses were conducted in RStudio 4.3.0 using the *stats* package (R Core Team, 2023).

## Results

### Categorization accuracy

**Figure 2.**
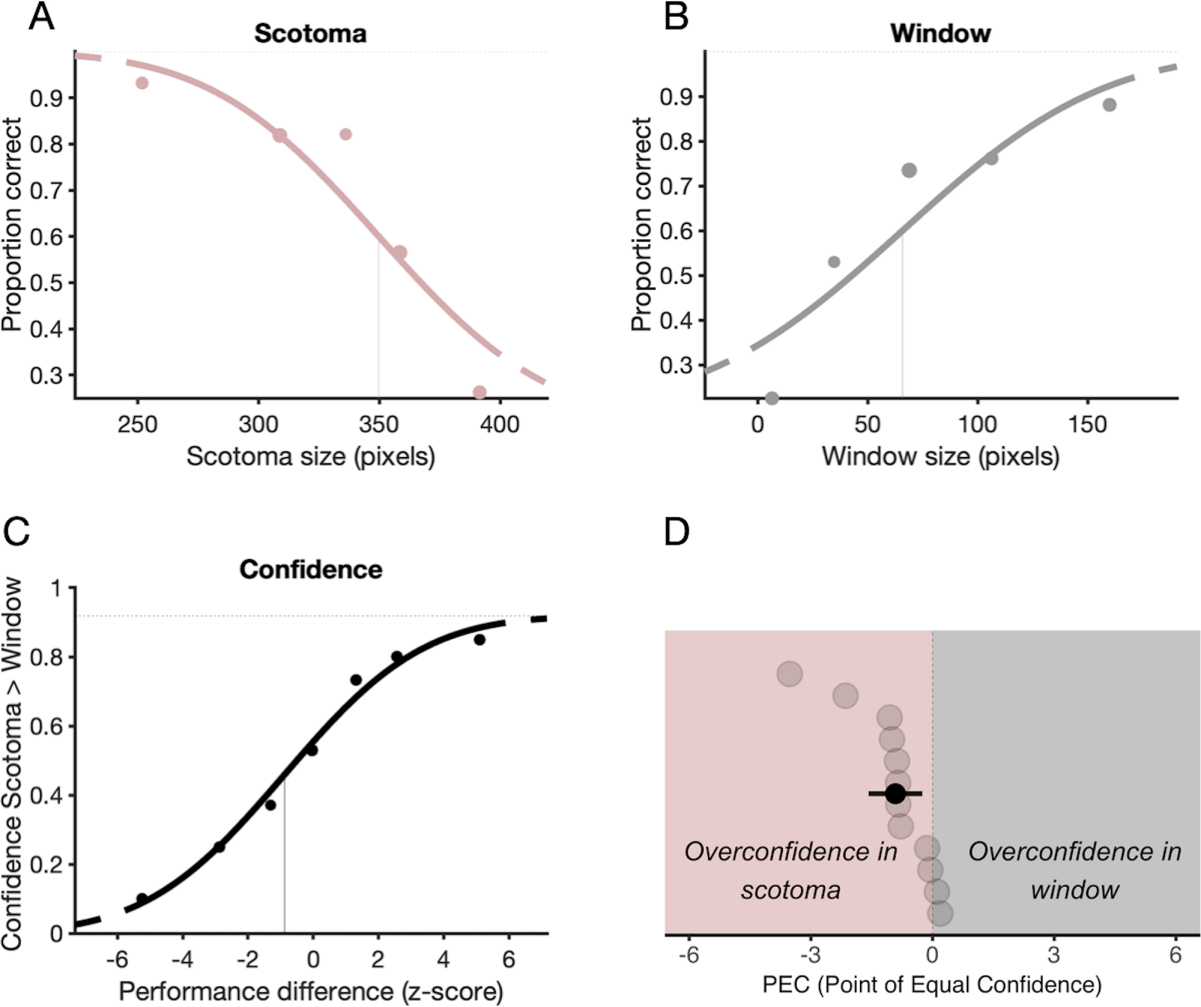
Accuracy and confidence results. (**A**) The psychometric function of one participant in the scotoma condition shows the proportion correct responses as a function of scotoma size in pixels. (**B**) The Psychometric function of the same participant in the window condition shows the proportion of correct responses as a function of window size in pixels. (**A**, **B**) Vertical lines indicate estimated thresholds at 50%. (**C**) The psychometric function of the same participant showing the proportion of more confidence in scotoma responses over performance difference between the two conditions in *z*-scores. The vertical line indicates a point of equal confidence (PEC). (**D**) Scatterplot of PEC values. The pink shaded area represents a region of negative PECs, indicating overconfidence in scotoma. The gray shaded area represents a region of positive PECs, indicating overconfidence in the window. Light gray dots show individual participant PECs, vertically ordered from lower to higher values; the black dot represents the mean PEC across participants. The error bar indicates the 95% confidence interval.

As expected, categorization accuracy decreased with increasing size of the scotoma (i.e., decreasing peripheral information) (Figure 2A) and increased with the increasing size of the window (i.e., increasing central information) (Figure 2B). The mean estimated threshold in scotoma was 370.89 ± 17.34 pixels (mean ± *SD*); in window, 67.19 ± 14.05 pixels (mean ± *SD*). In order to measure the efficiency of information extraction per unit in our image categorization task, we additionally calculated the ratio of the image area at the threshold radii for both the window and scotoma conditions. The window-to-scotoma ratio was 0.2601 ± 0.15 (mean ± *SD*), i.e. the image area at the thresholds in the window condition was smaller compared to the scotoma condition. This result indicates a higher efficiency of information extraction per unit in the center compared to the periphery.

### Metacognitive bias

The participants’ points of equal confidence were systematically shifted toward higher central perceptual performance (Figure 2D). The mean PEC was –0.915 (95% confidence interval (CI) [−1.578, −0.253]). On average, PEC was significantly different from zero, indicating overconfidence in the scotoma condition, *t*(11) = –3.0402, *p* = 0.011.

### Metacognitive sensitivity

**Figure 3.**
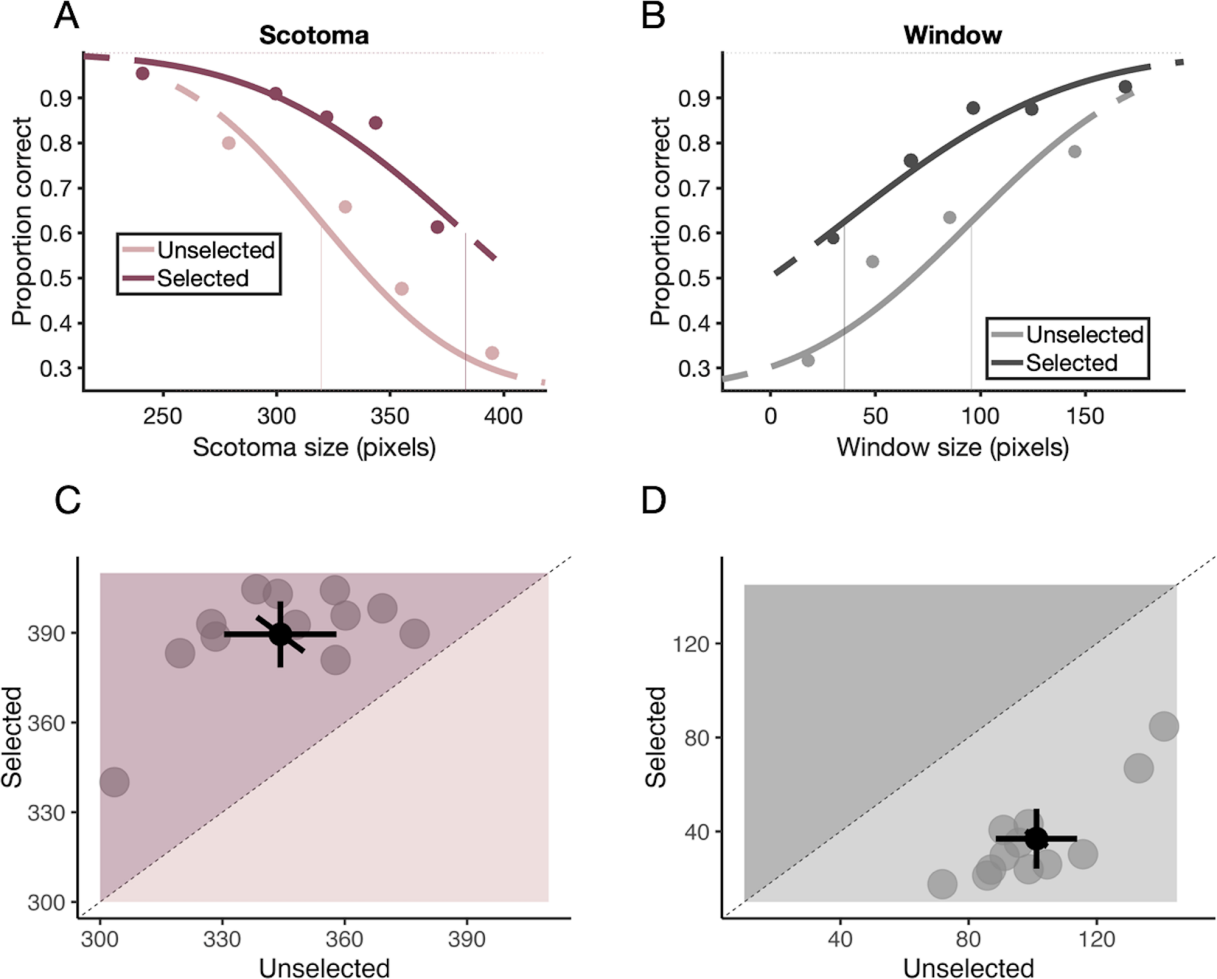
Metacognitive sensitivity results. (**A**) Psychometric functions of one participant for selected scotoma trials (higher confidence in scotoma) (dark pink) and unselected trials (lower confidence in scotoma) (light pink). (**B**) Psychometric functions of the same participant for selected window trials (higher confidence in window) (dark gray) and unselected trials (lower confidence in window) (light gray). (**A**, **B**) Vertical lines represent estimated thresholds at 50%. (**C**, **D**) Scatterplots of perceptual thresholds of individual participants (gray dots) with the overall means (black dots) in selected and unselected trials for scotoma (**C**) and window (**D**). Error bars indicate 95% confidence intervals.

In the trials where scotoma was selected with higher confidence (Figure 3C), the mean scotoma threshold was 389.49 ± 17.38 pixels (mean ± *SD*). In the trials where scotoma was not selected, it was 344.20 ± 21.69 pixels (mean ± *SD*). There was a significant difference between the selected and unselected scotoma trials, *t*(11) = –8.6504, *p* < 0.001, Cohen's *d* = 2.266; the mean difference was –45.293 (95% CI [−56.818, −33.769]). In the trials where window was selected with higher confidence (Figure 3D), the mean window threshold was 36.92 ± 20.03 pixels. In the unselected trials, it was 101.18 ± 19.94 pixels. There was a significant difference between the selected and unselected window trials, *t*(11) = 21.143, *p* < 0.001, Cohen's *d* = –3.216; the mean difference was 64.259 (95% CI [57.570, 70.948]). These results indicate that our participants produced confidence judgments that followed the perceptual performance in each trial and interval, rather than using solely window or scotoma size as a cue to confidence.

## Discussion

We combined a forced-choice confidence paradigm (Mamassian, 2020) with a scotoma/window scene categorization paradigm (Larson & Loschky, 2009). This allowed us to match categorization performance across peripheral and central conditions and isolate a metaperceptual bias. The results showed that the more scene information the observers were presented with, the better their categorization accuracy in both conditions. Their points of equal confidence were systematically shifted toward higher central perceptual performance. For equal confidence level, on average, higher central performance was needed to be matched with peripheral performance, suggesting more confidence in the periphery compared to the central vision (Figure 2D).

Although the tasks and stimuli used in our study and that of Toscani et al. (2021) are different, the underlying methodological approach is similar, allowing for a meaningful comparison between the two. Toscani et al. (2021) employed a fine-grained orientation discrimination task using Gabor patches, which relied on foveal processing. In contrast, our task required scene categorization, which depends more on global gist recognition mediated by peripheral vision. Whereas Toscani et al. (2021) found a metaperceptual bias favoring foveal information, we observed higher confidence in peripheral information. We speculate that this reversal in metaperceptual bias may have resulted from the shift in task demands from local, detail-oriented processing to global processing, emphasizing the role of task type in shaping metaperceptual judgments.

In the scotoma/window paradigm, peripheral stimuli need to cover a larger area of the visual field to yield equal performance as in central vision. Larson and Loschky (2009) found that, at the radius where the area sizes overlapped, the fovea showed superior accuracy. Our data also showed higher efficiency of extracting information per unit in the center compared to the periphery. Note that, because we used stimulus area to titrate performance, the paradigm takes into account the effects of cortical magnification (i.e., the fact that a larger cortical area is dedicated to processing of the foveal input). Increasing the amount of visual information in the periphery effectively compensates for the peripheral disadvantage (Larson & Loschky, 2009). On the other hand, this also introduces an additional difference between conditions. With regard to confidence in the scene categorization, observers may feel more confident because they perceive more of the stimulus in the periphery, even though they can extract less information per surface unit compared to the fovea. However, even if greater image area coverage contributes to greater confidence in the periphery, this characteristic is inherently linked to the nature of the peripheral vision. Our periphery covers a larger area of the visual field and thus specializes in the processing of global information. This means that controlling for image area across conditions would fail to capture characteristics of peripheral vision and would take away the key factor that contributes to superior peripheral scene recognition (see also Trouilloud et al., 2020).

What other factors might lead observers to have overconfidence in their peripheral view while categorizing scenes based on a partial view? Peripheral vision is often confronted with the problem of interpolating missing information over larger areas of the visual field. To support this process, different mechanisms exist, for instance perceptual filling-in, whereby missing visual information is illusorily reconstructed based on surrounding context and prior experience (for reviews, see Komatsu, 2006; Pessoa, Thompson, & Noë, 1998; Weil & Rees, 2011). This mechanism could create an illusion of perceiving more of the scene than what was actually presented, possibly through the spreading of the peripheral texture-like representation toward the central scotoma, in turn leading to higher confidence in peripheral content. Another possibility is amodal completion, wherein the visual system infers the presence of occluded surfaces or objects. This would involve interpreting the gray background as an occluder. However, since amodal completion typically relies on depth cues, which were absent in our experiment, this explanation becomes less likely. Moreover, one cannot rule out that similar processes leading to an inflated representation of the scene would be induced by the presentation of the central portion of a scene as well, as evidenced by the boundary extension phenomenon (Intraub & Richardson, 1989; for a review, see Hubbard, Hutchison, & Courtney, 2010). Although broader spatial coverage and filling-in mechanisms are characteristics of peripheral vision, the nature of the task and stimuli may also have influenced the results. Observers might have a tendency to rely on and eventually over-trust peripheral vision for categorizing scenes because scene-diagnostic features, or even scene-diagnostic objects, might tend to be localized closer to the borders of the scene image, at least for some scene categories. For example, the outline of the mountain profile against the sky in a mountain scene is unlikely to be located at the center of the view in a canonically taken photograph.

To better understand the observed overconfidence, we additionally assessed the metaperceptual sensitivity. The purpose was to make sure that observers’ confidence ratings followed their trial-by-trial accuracy, rather than relying only on the stimulus size cue for their confidence reports. The thresholds of selected and unselected confidence choices were systematically different, indicating high sensitivity for confidence in both conditions (Figures 3C and 3D). Given the complexity and variability built into our stimuli, it is reasonable to expect a higher level of confidence sensitivity as compared to the simpler stimuli used by Toscani et al. (2021). In Toscani et al. (2021), with their identical Gabors, performance fluctuations must be mostly due to internal noise (e.g., fluctuations in alertness), but our stimuli inherently contained some degree of noise, as some images would be more difficult to categorize than others. Nonetheless, the difference between selected and unselected stimuli is a clear indication that our participants were paying attention, had access to their performance accuracy, and selected intervals with higher confidence in which they also performed more accurately.

Previous research has shown both overconfidence and underconfidence in peripheral vision. Our data indicate that whether peripheral vision exhibits overconfidence or underconfidence largely depends on the specific task it is required to perform. The difference in confidence may depend on a concrete task dichotomy, whether the task involves simply detecting the presence of an object in the periphery (detection task) or identifying its details (discrimination task). Or, more generally, confidence could be predicted based on whether the periphery or fovea is typically used and thus is better suited for the task. Studies that have found overconfidence in peripheral vision have mostly included detection tasks (Li et al., 2018; Rahnev et al., 2011; Solovey et al., 2015). But, this is not always the case (e.g., the first experiment in Odegaard et al., 2018), and the distinction between detection and discrimination alone may not be the universal explanation for the occurrence of overconfidence and underconfidence. However, detecting objects in the environment is primarily a function of peripheral vision. In some cases, superior detection compared to discrimination performance has been observed at increasing eccentricities (Harris & Fahle, 1996; Levi, Klein, & Aitsebaomo, 1984). Consequently, it is plausible that confidence in detection is higher than in discrimination when relying on peripheral vision. Ziemba and Simoncelli (2021) showed a clear foveal–peripheral trade-off, where reduced detail discrimination in the periphery was compensated by an enhanced capacity for global processing. In the periphery, observers’ ability to discriminate fine detail in summary statistics diminishes as the size of stimuli increases, but the ability to discriminate stimuli with different statistics improves with larger stimuli. This specific superior discriminative ability of peripheral vision leaves room for speculation that there may be instances where overconfidence is observed in the periphery for specific discrimination tasks that are particularly suited to the characteristics of peripheral vision.

The statistical summary hypothesis (Balas, Nakano, & Rosenholtz, 2009; Ehinger & Rosenholtz, 2016; Rosenholtz, Huang, Raj, Balas, & Ilie, 2012) explains peripheral advantages in scene gist perception through spatial pooling models (Freeman & Simoncelli, 2011). Larger receptive fields in the periphery capture broad structures and textures, which are sufficient for recognizing basic scene categories (Loschky, Hansen, Sethi, & Pydimarri, 2010; Oliva & Torralba, 2006). The coarse-to-fine processing model of scene perception suggests that the peripheral advantage comes from the rapid processing of low spatial frequency information, which is primarily handled by peripheral vision and is sufficient for the gist extraction (Kauffmann, Chauvin, Guyader, & Peyrin, 2015; Trouilloud et al., 2020). Zhaoping (2024) refers to peripheral processing as “looking” to characterize the global, coarse selection guided by saliency and foveal detailed encoding as “seeing.” According to her, feedback verification is what mainly distinguishes peripheral and foveal vision. Peripheral vision excels at tasks such as monitoring the environment and perceiving the gist of the scene, largely because these tasks require minimal feedback verification. These tasks also align with the innate role of peripheral vision of guiding saccades and influencing decisions about when and where to shift the gaze and are usually performed with remarkable efficiency (Zhaoping, 2023; Zhaoping, 2024). Although we seem to be aware of the limitations of peripheral vision (e.g., Gloriani & Schütz, 2019; Kim & Chong, 2024; Pruitt et al., 2024; Toscani et al., 2021), in everyday life we do trust it to detect and respond to stimuli and orient ourselves in space (for a review, see Vater, Wolfe, & Rosenholtz, 2022). Accurate evaluation of the capabilities and limitations of our peripheral vision is crucial for the efficient distribution of limited processing resources. It would be costly not to trust the larger area of our visual processing with anything based on its limitations.

## Conclusions

We manipulated the amount of peripheral and central information using the scotoma/window paradigm to examine confidence in peripheral and central vision. Participants showed higher confidence in peripheral input when categorizing scenes, likely because the global processing needed for this task benefits from access to a wider visual field. In contrast, Toscani et al. (2021) observed underconfidence in the periphery when participants were asked to discriminate orientation. Together, these findings suggest that confidence may reflect not just perceptual efficiency but also the functional advantage of peripheral vision for certain tasks. This is likely shaped by prior experiences of relying on foveal versus peripheral vision for different tasks. In other words, we seem to be aware not only of the limitations of peripheral vision but also of its capabilities, indicating an implicit understanding of which tasks can and cannot be effectively handled by peripheral vision.
